# Salubrinal Regulates the Apoptosis of Adrenocortical Carcinoma Cells via the PERK/eIF2*α*/ATF4 Signaling Pathway

**DOI:** 10.1155/2021/5038130

**Published:** 2021-09-07

**Authors:** Lili Wu, Chunfeng Liang, Xuemei Huang, Xiujun Deng, Jiming Jiang, Zuojie Luo

**Affiliations:** ^1^Department of Endocrinology, The First Affiliated Hospital of Guangxi Medical University, Nanning, Guangxi 530021, China; ^2^Department of Integrated Medicine, Guangxi Medical University Cancer Hospital, Nanning, Guangxi 530021, China; ^3^Department of Analysis for Cosmetics (Dietary Supplements), Guangxi Institute for Food and Drug Control, Nanning, Guangxi 530021, China

## Abstract

The protein-kinase-*R-* (PKR-) like endoplasmic reticulum kinase (PERK) signaling pathway is a well-known promoter of cell apoptosis. In this study, we aimed to determine whether salubrinal (Sal), a selective activator of eukaryotic translation initiation factor 2 (eIF2*α*), can induce apoptosis of human adrenocortical carcinoma (ACC) cell via activating the PERK/eIF2*α*/ATF4 signaling pathway, and the potential mechanisms of this action were explored. The ACC cell lines, including SW-13 and NCI–H295 R, were used. 3-(4,5)-Dimethylthiazol(-z-y1)-3,5-di-phenytetrazoliumromide (MTT) assay, cell scratch experiments, flow cytometry, and JC-1 staining assays were performed to detect the cell viability, cell migration, and cell apoptosis. The expression of PERK/eIF2*α*/ATF4 signaling-pathway-related proteins and apoptosis-related proteins was detected by western blot (WB). Intracellular Ca^2+^ ion concentration was determined by a confocal laser scanning microscope. The results showed that Sal inhibited the migration and proliferation of ACC cells. Sal remarkably increased the influx of Ca^2+^ ion and the apoptosis rate of ACC cells *in vitro*. Furthermore, the expression levels of PERK/eIF2*α*/ATF4 signaling-related proteins and apoptosis-related proteins were upregulated in the treatment of Sal. The research demonstrated that Sal reduces the cell viability, increases the intracellular calcium concentration, and promotes the apoptosis of ACC cells *in vitro* through increasing the phosphorylation level of eIF2*α* and activating the PERK/eIF2*α*/ATF4 signaling. PERK/eIF2*α*/ATF4 is expected to act as a potential therapeutic target for the treatment of adrenocortical carcinoma.

## 1. Introduction

Adrenocortical carcinoma (ACC) is a kind of malignant tumor with low incidence but poor prognosis. ACC in most patients has already metastasized when the disease is diagnosed, and the risk of recurrence is very common after standard systemic therapies [1]. Currently, the most optimal strategy for adrenocortical cancer is surgery, but most patients have lost the opportunity for surgical treatment by the time they are diagnosed, and the cancer is often insensitive to radiotherapy and chemotherapy. Drug therapy has emerged as an effective therapeutic strategy. However, it still remained challenge due to the lack of effective therapeutic target. Therefore, to explore new therapeutic target for treatment of adrenocortical carcinoma is urgently needed. Extracted from human ACC tissue, the SW-13 cell line has been used for most of the basic research on anticancer drugs and cancer cell signaling pathways. The NCI–H295 R cell line, obtained from human ACC patient, has secretion function and is widely used in the study of ACC [2].

Endoplasmic reticulum (ER) stress is the main mechanism of apoptosis. The influencing balance of ER results in the production of unfolded proteins. Unfolded protein response (UPR) attempts to maintain ER homeostasis by activating a series of signaling pathways when unfolded proteins accumulated [3]. The three main signal pathways for maintaining ER homeostasis are PERK, ATF6 (activated transcription factor 6), and IRE1 (inositol-requiring enzyme 1). Of these three signaling mechanisms, the PERK signal pathway is activated first. The PERK/eIF2*α*/ATF4 signaling pathway is one of the most crucial pathways for the survival of cells exposed to a variety of stressors, such as toxic environments, malnutrition, and oxidative stress [4]. Active phosphorylation of eukaryotic translation initiation factor 2 (eIF2*α*) blocks the downstream protein translation of activating transcription factor 4 (ATF4). The active pathway involves the prevention of protein phosphatase 1 (PP1) to dephosphorylate p-eif2*α* [5]. CCAAT enhancer-binding protein homologous protein (CHOP) is the main downstream effector of the PERK pathway, and it functions as a proapoptotic factor [6].

Salubrinal (Sal), a selective activator of eIF2a, can enhance the phosphorylation of eIF2*α*, which leads to the expression of ATF4 [7]. Sal is also an inhibitor of the GADD34:PP1 holoenzyme complex which protects the neuronal cell through inhibition of eIF2alpha dephosphorylation and interrupts the other degenerative pathways [8]. Doxorubicin induces cholangiocarcinoma cell death through initiating ROS production and DNA damage, which could be aggravated by eIF2a inhibitor Sal [9]. However, whether the PERK signaling pathway is involved in Sal-induced apoptosis of ACC cells is still unclear.

Here, the role of Sal on the proliferation, migration, and apoptosis of ACC cells was explored. And whether Sal-activated PERK/eIF2*α*/ATF4 signaling pathway can inhibit the proliferation of ACC cells and promote the apoptosis of ACC cells was also investigated.

## 2. Materials and Methods

### 2.1. Reagents and Antibodies

Sal was purchased from Sigma Chemical Company (USA). Fetal bovine serum (FBS) was maintained from HyClone (USA). MTT and JC-1 were obtained from Beyotime (Shanghai, China). Antibodies against PERK, eIF2*α*, ATF4, Bcl-2, p-PERK, and p-eIF2*α* were obtained from Cell Signaling Technology (USA). Fluo-3/AM was purchased from Sigma Chemical Company (USA).

### 2.2. Cell Cultures

The human ACC cell lines (SW-13 and NCI–H295 R) were obtained from Shanghai Institute of Life Sciences (Shanghai, China). SW-13 cells were cultured in DMEM with 10% FBS and supplemented with 1 mm glutamine and 1% penicillin/streptomycin (Beyotime, China). NCI–H295 R cells were cultured in DMEM/F12 (Sigma-Aldrich, Japan) with 5% FBS and 1% penicillin/streptomycin. The NCI–H295 R cell medium also included 0.1% ITS (BD Biosciences, USA) as described previously [2]. All cells were cultured at 37°C and 5% CO_2_.

### 2.3. Cell Viability Assay

Cells were cultured with different concentrations (0, 50, 100, 150, and 200 *μ*m) of Sal for 12 h, 24 h, 36 h, and 48 h, respectively. The viability of the SW-13 and NCI–H295 R cells was determined by MTT according to the manufacturer's instructions as previously reported [10]. The same experiment was replicated three times.

### 2.4. Analysis of Cell Apoptosis

Cells were treated with 100 *μ*M of Sal for 24 h. Then, the cells were harvested and apoptotic cells were identified by flow cytometry following the manufacturer's instructions. The assessment was conducted for three times. The cell apoptosis rate was calculated using Cell Quest™ 3.0 software (BD, USA) as previously reported [11].

### 2.5. JC-1 Fluorescence Staining

The mitochondrial membrane potential was determined by the fluorescence probe, JC-1, a detection indicator in the early stage of apoptosis. Cells were treated with 100 *μ*m of Sal for 24 h. Then, the culture medium was removed, and the JC-1 dye was added to the working solution. The mitochondrial membrane potential of the cells was detected according to the manual of dye kit [12]. Images of the cells were taken using an inverted phase-contrast microscope (Nikon, Japan). The ratio of apoptotic cells to total cells in the visual field was calculated as the apoptotic rate.

### 2.6. Cell Migration Assay

Cell migration is a method to determine the ability of [13]. For the migration assay, SW-13 and NCI–H295 R cells were seeded at a density of 3 × 10^5^ cells/well in the plates and cultured with only conventional medium or with medium containing 100 *μ*m of Sal for 24 h. When cell confluence reached to approximately 90%, a scratch was made using a 200 *µ*I micropipette tip. The migration area was observed at 0 h, 24 h, and 48 h, and photos were obtained using optical microscopy (Leica, Germany). The wound area was analyzed with ImageJ software.

### 2.7. Western Blot Analysis

Experimental cells were treated with 100 *μ*m of Sal for 24 h. The cells were harvested and lysed by RIPA buffer (Beyotime, China) for one-half hour. Fifty micrograms of total extracted cell protein was added to each gel lane for SDS-PAGE and then transferred to a PVDF membrane and blocked with 5% nonfat milk for 1 h which had been dissolved in TBST at RT as previously described [14]. The membrane was incubated overnight at 4°C with primary antibodies against PERK, p-PERK, eIF2*α*, p-eIF2*α*, ATF4, and Bcl-2 (all 1 : 1000 dilution). The next day, the membranes were incubated with secondary antibodies and visualized with a LI-COR platform (Odyssey, USA). GAPDH (CST, USA) was used as internal control. ImageJ software was used to measure the gray values of the proteins.

### 2.8. Statistical Analysis

Data are presented as the mean ± standard deviation (SD). Graphs were generated by GraphPad Prism 6.0. All statistical analyses were performed using SPSS 22.0. Differences between two groups were determined by unpaired Student's *t*-tests. ^*∗*^*p* < 0.05; ^*∗∗*^*p* < 0.01; and ^*∗∗∗*^*p* < 0.001 indicate significance.

## 3. Results

### 3.1. Cell Line Characterization

**Figure 1:**
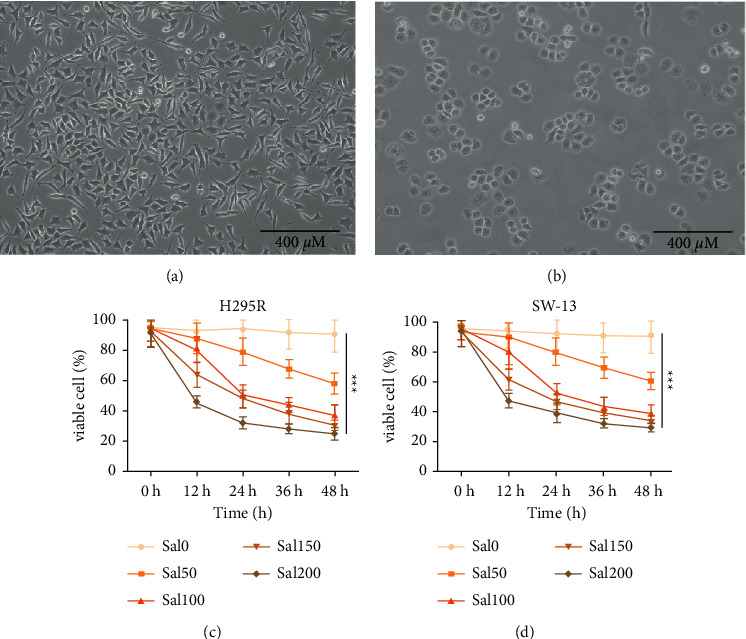
Cell line identification and the effect of Sal on the viability of ACC cells. (a, b) The cellular morphology of ACC NCI–H295 R and SW-13 cells (10×, scale bar is 400 *μ*m). (c, d) H295 R and SW-13 cells were cultured with Sal at concentrations of 0, 50, 100, 150, and 200 *μ*m for 0, 12, 24, 36, and 48 h. Each experiment was repeated at least 3 times. ^*∗∗∗*^*p* < 0.001.

NCI–H295 R cells were spindle in shape and showed characteristics of hyper chromatic nuclei (Figure 1(a)), while SW-13 cells were round in shape and with hyper chromatic nuclei (Figure 1(b)).

### 3.2. Sal Reduced the Cell Viability of ACC Cells

Cells treated with different concentrations of Sal (0 *μ*m, 50 *μ*m, 100 *μ*m, 150 *μ*m, and 200 *μ*m) for different durations (12 h, 24 h, 36 h, and 48 h) were measured with MTT assays. As shown in Figures 1(c) and 1(d), 50 *μ*m, 100 *μ*m, 150 *μ*m, and 200 *μ*m of Sal significantly decreased the cell viability of the ACC cells treated with Sal for 24 h, 36 h, and 48 h (*p* < 0.05, compared to control). The one-half maximal inhibition concentration of Sal in the NCI–H295 R and SW-13 cells were 104.177 *μ*m and 102.341 *μ*m, respectively. Sal significantly decreased the cell viability of the ACC cells when the cells were treated with Sal for 24 h (the viability of SW-13 was 52.34 ± 7.03% in 100 *μ*m of Sal intervention group and 92.65 ± 14.27% in the control group; the viability of H295 R was 51.06 ± 6.45% in the 100 *μ*m Sal intervention group and 94.13 ± 13.79% in control group) (*p* < 0.05 compared to control). Sal has no significant effect on ACC cells at low does (<75 *μ*m) (Figure S1). Therefore, we used Sal at a concentration of 100 *μ*m in subsequent investigations.

### 3.3. Sal Inhibits the Migration of ACC Cells

**Figure 2:**
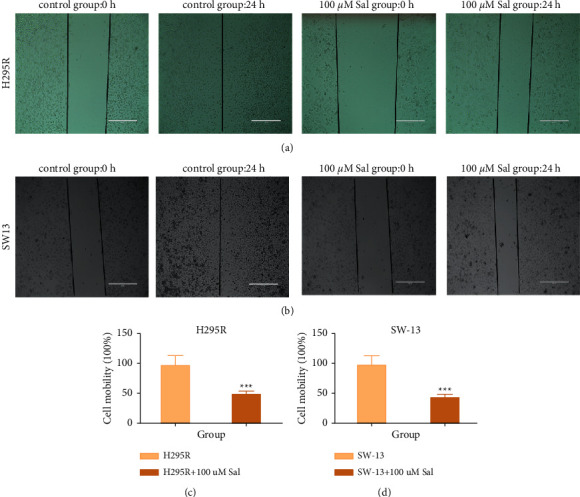
The effect of Sal in inhibition of the migration of ACC cells. (10×, scale bar is 400 *μ*m). (a, b) Cell migration of H295 R and SW-13 cells after being incubated with 0 *μ*m Sal or 100 *μ*m Sal for 0 h and 24 h. (c, d) Calculation of cell migration of H295 R and SW-13 cell in A and B; ^*∗∗∗*^*p* < 0.001.

To explore the role of Sal in migration of ACC cells, wound healing assay was carried out. It demonstrated that incubation with 100 *μ*m of Sal for 24 h significantly inhibited the migration capacity of the ACC cells (SW-13 and H295 R), while inverse results were observed in a routine culture of ACC cells (Figures 2(a) and 2(b)). Sal significantly decreased the cell migration of the ACC cells when the cells were treated with Sal for 24 h (the migration of SW-13 is 40.17 ± 6.82% in 100 *μ*m of Sal intervention group and 97.24 ± 15.45% in control group; the migration of H295 R is 47.39 ± 6.18% in 100 *μ*m Sal intervention group and 96.72 ± 17.58% in control group) (*p* < 0.001 compared to control) (Figures 2(c) and 2(d)).

### 3.4. Sal Induces Apoptosis of Human ACC Cell

**Figure 3:**
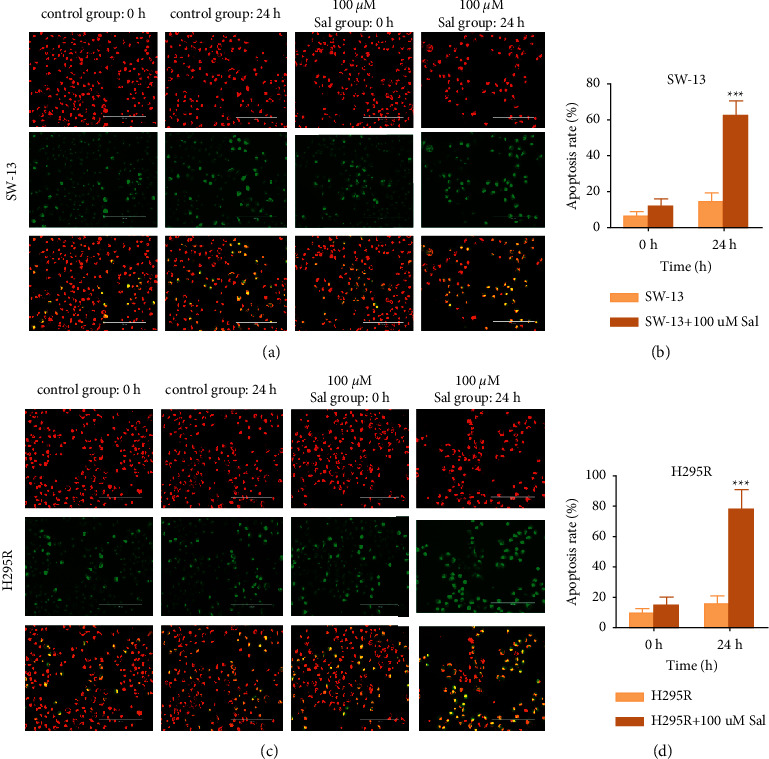
Sal induced apoptosis in ACC cells. (a) SW-13 cells were stained with JC-1 after being incubated in 0 *μ*m Sal and 100 *μ*m Sal for 0 h and 24 h. (b) Comparison of cell apoptosis in A ^*∗∗∗*^*p* < 0.001. (c) H295 R cells were stained with JC-1 after being incubated in 0 *μ*m Sal and 100 *μ*m Sal for 0 h and 24 h. (d) Comparison of cell apoptosis in C ^*∗∗∗*^*p* < 0.001. The data are expressed as means ± SD (*n* = 3).

To investigate whether the ACC cells underwent apoptosis in the treatment of Sal, flow cytometry was performed to detect the apoptosis rate, WB was used to detect the expression of the apoptosis-related protein Bcl-2, and JC-1 staining was used to detect changes in the in-mitochondrial membrane potential of the cells to confirm the occurrence of apoptosis. As shown in Figures 3(a) and 3(c), the results of JC-1 staining revealed that the color of the cells staining changed from red to green, indicating changes to the mitochondrial membrane potential. Therefore, the cells underwent apoptosis, and the apoptosis rate of SW-13 is 12.31 ± 7.81% (0 h) and 62.45 ± 6.52% (24 h) in 100 *μ*m of Sal intervention group and 6.37 ± 2.45% (0 h) and 14.57 ± 4.66% (24 h) in control group; the apoptosis of H295 R is 14.96 ± 5.13% (0 h) and 78.32 ± 12.76% (24 h) in 100 *μ*m of Sal intervention group and 9.46 ± 3.14% (0 h) and 15.89 ± 5.24% (24 h) in control group. Significant differences were detected in apoptosis rates in the Sal intervention group and control group after 24 h (*p* < 0.001) (Figures 3(b) and 3(d)).

**Figure 4:**
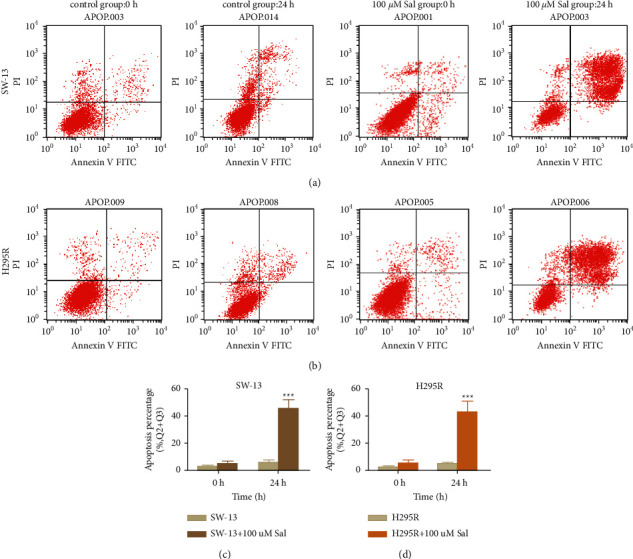
The effect of Sal in apoptosis in ACC cells. (a, b) The apoptosis rate of the ACC cells incubated in 0 *μ*m or 100 *μ*m of Sal for 0 h and 24 h. (c, d) Comparison of cell apoptosis rate of SW-13 and cells in A and B. The data represent the means ± SD (*n* = 3). ^*∗∗∗*^*p* < 0.001 compared with the control group.

The rate of apoptosis in the Sal-treated cells was further evaluated by flow cytometry. As shown in Figure 4, the apoptosis rate of the cells in the Sal (100 *μ*m) treatment group for 24 h was significantly higher than that of the nonintervention group (the apoptosis rate in SW-13 is 45.67 ± 6.48% in 100 *μ*m of Sal intervention group and 5.78 ± 1.74% in control group; the apoptosis rate in H295 R is 43.49 ± 7.44% in 100 *μ*m Sal intervention group and 4.82 ± 1.03% in control group).

**Figure 5:**
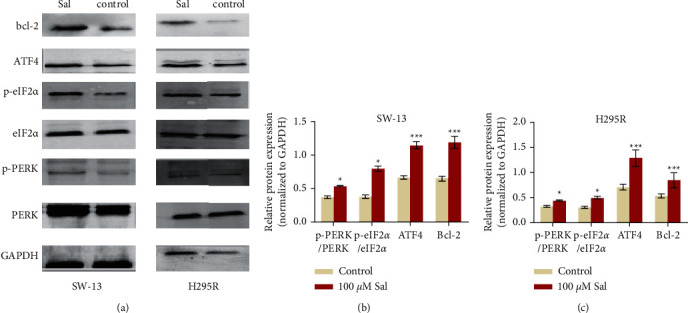
Effect of Sal in the expression of apoptotic and PERK/eIF2*α* signaling-related proteins. (a) Expression of Bcl-2 and the PERK/eIF2*α* signaling-related proteins PERK, p-PERK, eIF2*α*, p-eIF2*α*, ATF4 in SW-13 and NCI-H295 R cells, after treatment with 100 *μ*m of Sal or not for 24 h. (b, c) Quantification of the expression levels of Bcl-2 and the PERK/eIF2*α* signaling-related proteins PERK, p-PERK, eIF2*α*, p-eIF2*α*, and ATF4 in A. The data are expressed as means ± SD (*n* = 3). ^*∗*^*p* < 0.05, ^*∗∗*^*p* < 0.001, and ^*∗∗∗*^*p* < 0.001 compared with control group.

Bcl-2 family proteins are associated with the process of apoptosis, and the change in Bcl-2 protein level indicates the occurrence of apoptosis. Therefore, to compare the incidence of apoptosis in the two groups, the change in the Bcl-2 protein level was detected. The results showed that the Bcl-2 protein in the Sal (100 *μ*m) treatment group was significantly inhibited, indicating that the rate of apoptosis was significantly increased due to Sal (100 *μ*m) treatment as compared with the control group (Figures 5(a)–5(c)).

### 3.5. Sal Induces ACC Cell Apoptosis via the PERK Pathway

To analyze whether the PERK/eIF2*α* signaling pathway was involved in the mechanism of Sal-induced ACC cell apoptosis, the protein levels of p-PERK, p-eIF2*α*, and ATF4 were measured via WB. At a concentration of 100 *μ*m, Sal markedly increased the protein expression levels of p-PERK, p-eIF2*α*, and ATF4 in the two lineages of ACC cells (*p* < 0.05) (Figures 5(a)–5(c)).

## 4. Discussion

In this study, we investigated the role of Sal in the regulation of the proliferation, invasion, migration, and apoptosis of ACC cells through regulating the PERK/eIF2*α* signaling pathway. The results indicated that Sal inhibited the proliferation, invasion, and migration and induced the apoptosis of ACC cells by upregulating the PERK/eIF2*α* signaling pathway.

It is generally accepted that changes in the cellular environment, such as the induction of oxidative stress, cytotoxicity, and tumorigenesis, can lead to cell metabolism disorders, as indicated by increases in endoplasmic reticulum (ER) stress and unfolded protein response (UPR). UPR either promote stability of the intracellular environment or initiate a cell death process [15]. In our previous studies, the ER was found to play an important role in apoptosis of ACC cells [16]. The UPR is based on three important ER stress-related proteins: PERK, IRE1, and ATF6.

PERK is the most important sensor for ER stress. When ER stress occurs, it activates ATF4 and protein translation to enable cells to adapt to the new environment and undergo self-repair. PERK signaling is based on activating phosphorylated PERK to activate the downstream translation promoter eIF2*α*, which blocks most protein translation, including ATF4.

Under ER stress, eIF2*α* is phosphorylated by eIF2 kinases and inhibits protein translation. In addition, p-eIF2*α* induces the stress response by activating signaling downstream of ATF4 [17]. Of the four proteins (PERK, GCN, PKR, and HRI) known to affect eIF2*α*, PERK has the greatest activation effect [18]. The eIF2*α*–ATF4 pathway not only maintains the stability of the intracellular redox environment but also regulates cellular metabolism and nutrient uptake [4, 19]. This pathway is also important for the adaptation of tumor cells in a hypoxic microenvironment and contributes to tumor growth [20] and the development of resistance to chemotherapy drugs [21–23].

Sal, a selective activator of eIF2a, can prevent eIF2*α* dephosphorylation by inhibiting the formation of protein complex GADD34/PP [24, 25]. It has been reported that Sal showed anticancer properties in cancer cells by manipulating the endoplasmic reticulum stress pathway or through promoting DNA damage [9, 26]. It also demonstrated that it could offer neuroprotection through UPR-related signaling factors along with other biochemical events [8]. The concentrations of Sal utilized in different organs or tissues showed varying difference [27–29]. In the present study, we have observed that the viability of the ACC cells was inhibited by Sal at the given dose. Sal had no significant effect on ACC cells at low does (<75 *μ*m) (Figure S1). The major reason is supposed to be that the concentration of Sal action in different organ cells is different [30].

Bcl-2 family proteins are important in regulating apoptosis signaling pathways [31]. Overexpression of Bcl-2 makes cells resistant to apoptosis, a phenomenon found in breast cancer and many other cancers [32]. Our results show that Bcl-2 was decreased by Sal in adrenocortical carcinoma cells (Figure 5), a finding that is consistent with previous research.

Sal inhibited the proliferation (Figure 1) and migration (Figure 2) of SW-13 and NCI–H295 R cells. The migration of H295 R cells is different from SW13 since the growth of H295 R cells is Spindle shaped and foot shaped. Furthermore, Sal promoted SW-13 and NCI–H295 R cell apoptosis (Figures 3–5). Yu et al. [9] revealed that Sal can promote ROS-mediated DNA damage to enhance the sensitivity of cells to doxorubicin to induce apoptosis in cholangiocarcinoma cells; similar results were obtained in this study. Some studies have shown that Sal can protect cells from the endoplasmic reticulum (ER) stress-induced apoptosis [24], which is somewhat inconsistent with the results of this study. One possible reason for this finding is that Sal added at low concentrations can have a protective effect on cells, while Sal added at high concentrations promotes apoptosis.

Cells protrude through the front end, locally form focal spots, surrounding hydrolyzed fibers, cell body contraction, and rear end separation to complete a migration cycle. Formation of cell protrusion (also known as pseudopod) is an essential step for cell migration. The formation of protrusion mainly depends on the flow of actin or the polymerization of actin fibers, the contraction of the cytoskeleton, and increased concentration of calcium ions and Rho GTPase. Focal adhesion between cells induces movement and cell advancement by transmitting sliding motion of actin stress fiber bundles, thereby exerting signal and mechanical conduction functions [33–35]. Podosomes are other structures which play an important role in cell migration while Ca^2+^ signaling is essential in the assembly of podosomes. Our results show that Sal diminished cell migration in ACC cells (Figure 2). Indicating Sal may inhibit cell migration through increased cell apoptosis.

The present study revealed that Sal could inhibit the proliferation of ACC cells by inducing apoptosis. Additionally, Sal suppresses the expression of apoptosis-related protein Bcl-2 through activated PERK/eIF2*α*/ATF4 signaling pathway.

## 5. Conclusion

In summary, the sensitivity of ACC cells to Sal is related to the regulation of apoptosis by activating the PERK/eIF2*α*/ATF4 signaling pathway.

## Data Availability

The data are available upon request (data contact details: wuliligxmu@126.com).

## Abbreviations

Sal: Salubrinal
PERK: Protein-kinase-*R-* (PKR-) like endoplasmic reticulum kinase
eIF2*α*: Eukaryotic translation initiation factor 2
ATF4: Activated transcription factor 4
ACC: Adrenocortical carcinomas
ER: Endoplasmic reticulum
MTT: 3-(4,5-dimethylthiazol-2-yl)-2,5-diphenyl tetrazolium bromide
JC-1: Mitochondrial membrane potential assay kit with JC-1
CLSM: Confocal laser scanning microscope.

## Supplementary Materials

Supplementary Materials Figure S1. flow cytometry was used to explore the apoptosis rate of the ACC cells induced by low concentrations of Sal. The apoptosis rate of SW-13 and H295 R cells was detected after being incubated with 0 *μ*m, 5 *μ*m, 10 *μ*m, 15 *μ*m, 20 *μ*m, 25 *μ*m, 50 *μ*m, or 75 *μ*m Sal for 24 h.
