# Supplementary material for: Salubrinal Regulates the Apoptosis of Adrenocortical Carcinoma Cells via the PERK/eIF2α/ATF4 Signaling Pathway
Source: Int J Endocrinol. 2021 Sep 7;2021:5038130. doi: 10.1155/2021/5038130 (PMC8461226; doi:10.1155/2021/5038130)
Supplement: Supplementary Materials — Figure S1. flow cytometry was used to explore the apoptosis rate of the ACC cells induced by low concentrations of Sal. The apoptosis rate of SW-13 and H295 R cells was detected after being incubated with 0 μm, 5 μm, 10 μm, 15 μm, 20 μm, 25 μm, 50 μm, or 75 μm Sal for 24 h. [file 5038130.f1.docx]

Figure S1.


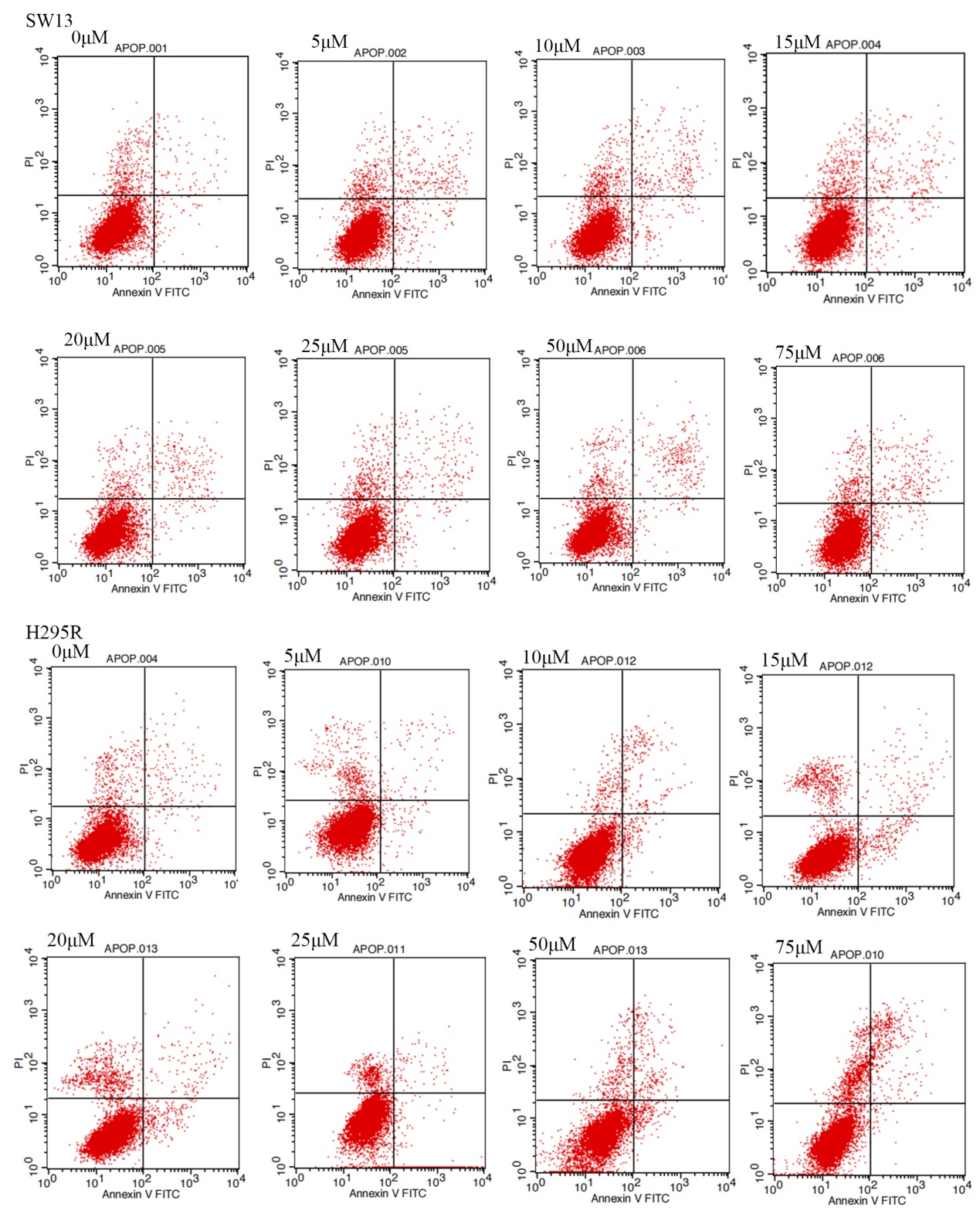


**Figure S1.** Flow cytometry was used to explore the apoptosis rate of the ACC cells induced by low concentrations of Sal. The apoptosis rate of SW-13 and H295R cells was detected after incubated with 0 µM, 5 µM, 10 µM, 15 µM, 20 µM, 25 µM, 50 µM or 75 µM Sal for 24 h.
